# Mirror neuron brain regions contribute to identifying actions, but not intentions

**DOI:** 10.1002/hbm.26036

**Published:** 2022-07-30

**Authors:** Emma L. Thompson, Geoffrey Bird, Caroline Catmur

**Affiliations:** ^1^ Department of Psychology, Institute of Psychiatry, Psychology and Neuroscience King's College London London UK; ^2^ Department of Clinical and Health Psychology University of Edinburgh Edinburgh UK; ^3^ Social, Genetic and Developmental Psychiatry Centre, Institute of Psychiatry, Psychology and Neuroscience King's College London London UK; ^4^ Department of Experimental Psychology University of Oxford Oxford UK

**Keywords:** action perception, action understanding, functional magnetic resonance imaging, mirror neuron, transcranial magnetic stimulation

## Abstract

Previous studies have struggled to determine the relationship between mirror neuron brain regions and two distinct “action understanding” processes: identifying actions and identifying the intentions underlying those actions. This may be because the identification of intentions from others' actions requires an initial action identification process. Disruptive transcranial magnetic stimulation was administered to left inferior frontal gyrus (lIFG) during a novel cognitive task to determine which of these “action understanding” processes is subserved by mirror neuron brain regions. Participants identified either the actions performed by observed hand actions or the intentions underlying those actions. The extent to which intention identification was disrupted by lIFG (vs. control site) stimulation was dependent on the level of disruption to action identification. We subsequently performed functional magnetic resonance imaging during the same task. During action identification, responses were widespread within mirror neuron areas including lIFG and inferior parietal lobule. However, no independent responses were found in mirror neuron brain regions during intention identification. Instead, responses occurred in brain regions associated with two distinct mentalizing localizer tasks. This supports an account in which mirror neuron brain regions are involved in an initial action identification process, but the subsequent identification of intentions requires additional processing in mentalizing brain regions.

## Graphical abstract

The involvement of mirror neuron brain regions in action understanding has been difficult to determine. Two experiments using transcranial magnetic stimulation and functional magnetic resonance imaging demonstrated that mirror neuron regions are involved in action identification but not in identification of others' intentions. This suggests that mirror neuron brain regions are involved in an initial action identification process, but the subsequent identification of intentions requires additional processing in mentalizing brain regions.

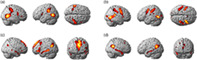

When observing someone else's actions we need to know not only what they are doing, but why they are doing it. It has been suggested that identifying the intention that underlies another person's action is performed by “mirror” neurons (Fogassi et al., 2005; Rizzolatti & Fogassi, 2014), but this conclusion is controversial (Heyes & Catmur, 2022), with other evidence suggesting that identification of others' intentions relies on “mentalizing” brain regions (Brass et al., 2007). Previous attempts to address this issue have been impacted by factors such as stimulus confounds, alongside difficulties in separating out the cognitive and neural processes that support intention identification from those that are involved in action identification. The present study reports the results of one neurostimulation and one neuroimaging experiment which seek to establish the extent to which mirror neuron brain regions are involved in identifying the intentions underlying others' actions.

Mirror neurons were first reported in the macaque monkey, in ventral premotor cortex (di Pellegrino et al., 1992). Neurons with similar firing patterns have since been recorded in other species including in humans (Mukamel et al., 2010), and there are various noninvasive recording techniques which support the claim that similar neurons exist in homologous areas of the human brain (Fadiga et al., 1995; Molenberghs, Cunnington, et al., 2012). Mirror neurons fire primarily when the animal is performing an action. However, they also fire when the animal merely observes someone else performing the same action. This response pattern suggests that mirror neurons map an observed action onto the observer's own motor programme for that action, and this has led some researchers to claim that mirror neurons allow the observer to understand others' actions “from the inside” (Rizzolatti & Sinigaglia, 2010), and thus that mirror neurons support the “understanding of actions made by others” (Rizzolatti et al., 1999). However, the term “action understanding” has been used to refer to at least two cognitive processes, which correspond to *action identification*, differentiating an observed action from other similar actions; and *intention identification*, determining the intention that underlies the observed action (Catmur, 2014; Thompson et al., 2019); and it is not clear which of these two processes is subserved by mirror neurons.

In an influential early study investigating the role of mirror neuron brain regions in intention identification, Iacoboni et al. (2005) found that the right inferior frontal cortex showed a greater response to actions when performed in certain contexts, which provided information about the actor's intentions, than to actions presented without contextual cues. However, the original contrast was confounded by the fact that the stimuli were not matched for the number of objects they contained. Differential activity of motor cortical areas in the intention condition may therefore have been the result of the objects' motor affordances (Grèzes et al., 2003; Mecklinger et al., 2004) rather than reflecting anything to do with intention identification. This type of confound makes it difficult to interpret the results of neuroimaging studies in which the action identification and intention identification conditions are not well matched. Even when conditions are matched for the presence of objects, in some tasks the intention identification task requires object processing while the action identification task does not (Spunt & Adolphs, 2014), again producing a situation where brain responses to intention identification are conflated with those for object processing.

A further problem arises when the processes of interest (i.e., action vs. intention identification) are confounded with lower‐level features of the stimulus, for example kinematic differences (Becchio et al., 2014). In neuroimaging studies it is difficult to distinguish between differences in brain responses to intention versus action identification, and differences that result from the kinematic differences in the stimuli.

Finally, Spunt et al. (2016) demonstrated that answers to action identification questions (“what is she doing?”) are less abstract than answers to intention identification questions (“why is she doing it?”), and that furthermore such differences in abstraction, even in the absence of action stimuli, are associated with responses in mirror neuron brain regions.

Therefore, any study which aims to distinguish action identification from intention identification needs to ensure that these two task conditions are matched in terms of the presence of objects (and their involvement in the two processes); any low‐level stimulus features such as kinematics; and the level of abstractness of the stimuli and any associated questions.

Another difficulty that has undermined efforts to dissociate neural substrates for action and intention identification is that intention identification may require action identification as an initial process. That is: in order to identify the intention underlying an action, one must first identify the action that is being performed. Thus brain areas which respond during intention identification may be contributing to the initial action identification process, the subsequent intention identification process, or both. This makes it difficult to interpret the results of neurostimulation experiments which claim to show that disruption of mirror neuron brain regions impacts intention identification (e.g., Michael et al., 2014): any disruption to intention identification may be the result of disrupting the initial action identification process (Catmur, 2014). As a result, in order to determine that a brain region contributes to intention identification, neuroimaging studies need to demonstrate that it contributes over and above its contribution to action identification; and neurostimulation studies need to demonstrate that disruption to intention identification is additional to any disruption to action identification.

In this article we present both a neurostimulation and a neuroimaging experiment using a new task which fulfils the recommendations specified above (Thompson, Bird, et al., 2022) in order to determine whether mirror neuron brain regions contribute to action or intention identification. The hand action task includes two conditions: the *action identification* condition targets the identification of the configuration of the body parts involved in the observed action; and the *intention identification* condition targets the identification of the intention underlying the observed action. A range of variables are controlled across the action and intention conditions, including the level of abstractness and the stimuli themselves (e.g., by removing object processing demands and kinematic differences). A control task is also included to control for non‐specific processing demands.

In Experiment 1 we sought to determine whether a key mirror neuron brain region, left inferior frontal gyrus (lIFG), is involved in identifying actions and/or the intentions underlying those actions, by disrupting lIFG and a control brain site with repetitive transcranial magnetic stimulation (rTMS) during action and intention identification. lIFG was chosen as it responds consistently during mirror neuron localiser tasks and also when observers are determining the goal of observed body movements (van Overwalle & Baetens, 2009). Brain stimulation studies also support the involvement of this area in identifying intentions underlying observed actions (Michael et al., 2014; Tidoni et al., 2013).

If lIFG is involved in action identification, then response times to identify actions should be slower, and/or accuracy to identify actions should be lower, following lIFG stimulation compared to control site stimulation. If lIFG is independently involved in intention identification then we should see an additional effect of lIFG stimulation on intention identification (i.e., greater response time slowing/reduction in accuracy than what is seen for action identification). In contrast, if the contribution of lIFG to intention identification is purely as a result of its contribution to action identification, then any effect of lIFG stimulation on intention identification should be explained by the level of disruption to action identification (e.g., disruption to action identification, when used as a covariate, should remove any effect of stimulation site on intention identification; and the effect of lIFG stimulation on action identification should predict its effect on intention identification).

Intentions may be identified somewhat later than actions (e.g., Thompson, Long, et al., 2022 found intentions were identified c. 30 ms slower than actions). This is consistent with our suggestion that action identification contributes to intention identification: if action identification contributes to intention identification then it has to be the case that intentions are processed later than actions; and any disruption of action identification should impact on intention identification. Alternatively, it is possible that action and intention identification are independent processes that take place at different times; in which case, intentions might still be processed later than actions but any disruption to action identification should not impact on intention identification. Accordingly we included a range of stimulation times in order to test whether the impact of lIFG stimulation on action and/or intention identification varied as a function of time after stimulus presentation.

## 1 EXPERIMENT 1

### 1.1 Method

#### 1.1.1 Participants

A power analysis conducted using G* Power 3 (Faul et al., 2007) indicated a minimum sample size of 34 participants to detect a medium effect size for the interaction of site and task, with a power of over .8 at an alpha level of .05. Thirty‐six right‐handed native English speakers (seven males) aged 18–46 years (mean = 24.03, SD = 5.16) were therefore recruited via King's College London recruitment email. Participants had no contraindications for TMS (Rossi et al., 2009). Participants for both experiments had no history of neurological or psychiatric disorders and were compensated a small fee for their time. Experimental procedures for both experiments were approved by the King's College London Research Ethics Committee and were carried out in accordance with the Declaration of Helsinki.

#### 1.1.2 Design

Participants received disruptive stimulation of the lIFG and the vertex, in counterbalanced order, while performing an action understanding task and a control task, again in counterbalanced order. Each task comprised two conditions, action identification and intention identification, which were randomly distributed throughout the task.

#### 1.1.3 Stimuli

Sixteen images were used within each task (action understanding: hand images, and control: appliance images). For each task, each image was presented with both an action word phrase and an intention word phrase, and thus each condition comprised 16 word phrase‐image stimuli. Each image was presented twice in each condition (once with a matching and once with a mismatching word phrase), resulting in a total of 32 trials per condition; 64 trials per task. Trials from each condition were randomly distributed throughout the task.

**FIGURE 1:**
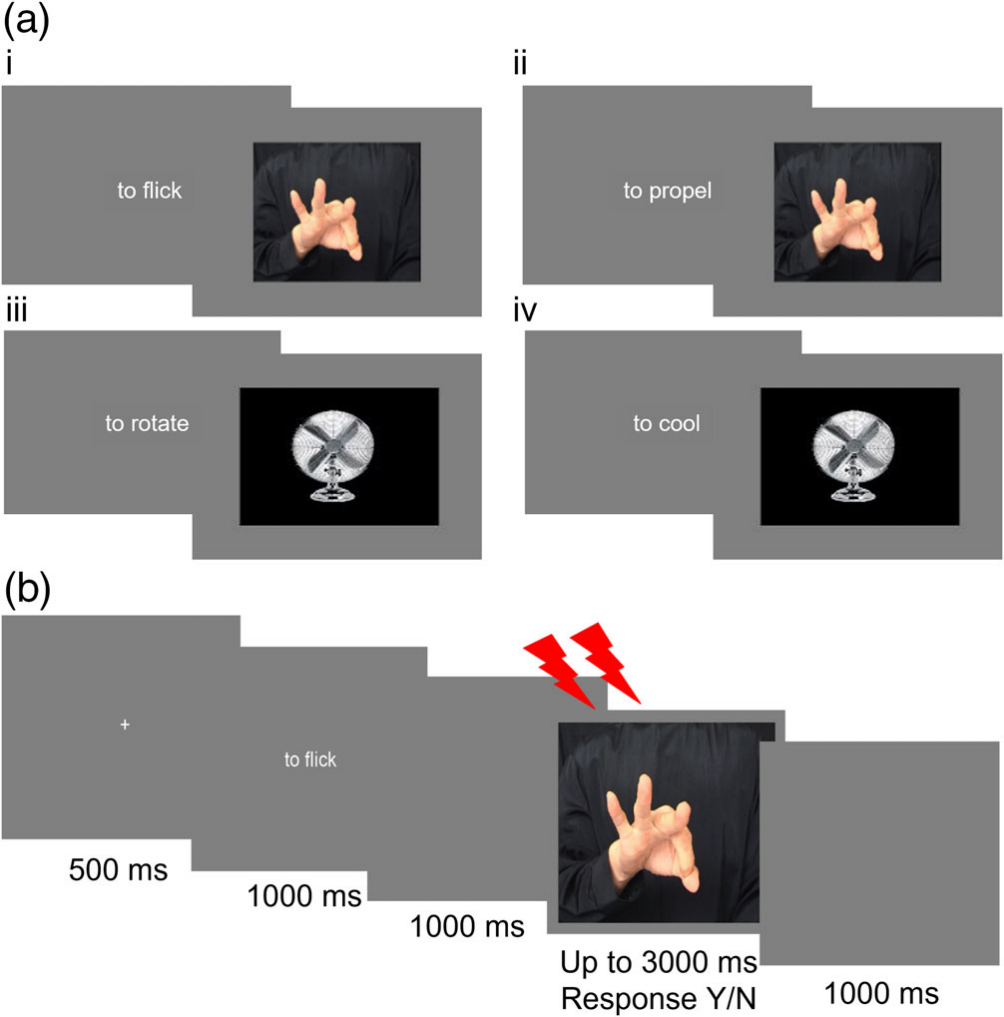
(a) Illustrative stimuli for action understanding and control tasks completed in experiment 1. (i) Hand action identification; (ii) hand intention identification; (iii) appliance action identification; (iv) appliance intention identification. (b) TMS trial procedure. Participants were required to indicate whether the word phrase (describing the action, e.g., “to flick”; or the intention, e.g., “to propel”) matched (response = yes) or did not match (response = no) the image

Hand stimuli consisted of still images depicting pantomimed hand actions, adapted from video clips created by Molenberghs, Hayward et al. (2012; see Figure 1). Each image was assigned two corresponding word phrases: one relating to the configuration of hand parts depicted in the image (action identification), and the other relating to the motivation underlying the depicted action (intention identification). The action and intention identification conditions were therefore fully matched in terms of the image properties. Each word phrase consisted of two to three words starting with the word “to”, for example, “to turn” (action) or “to open” (intention). The word phrases in the action and intention conditions were matched on a range of variables including: number of words (*t*[15] = 1.46, *p* = .164); number of characters (*t*[15] = 0.24, *p* = .812); word frequency, as determined by per million words in the SUBTLEX database (van Heuven et al., 2014), *t*(15) = 0.80, *p* = .435, and abstractness ratings (Brysbaert et al., 2014), *t*(15) = 0.76, *p* = .457.

The control, appliance, task was structured in the same way as the action understanding task, but stimuli comprised images of household appliances. Each image was assigned two word phrases, one relating to the action performed by the object (action identification), and the other relating to the purpose of the object (intention identification); for example, “to rotate” or “to cool”, describing a rotary fan. The word phrases in the action and intention conditions were matched on the same variables: number of words (all consisting of two words), number of characters *t*(15) = 0.36, *p* = .723, word frequency *t*(15) = 0.85, *p* = .410 and abstractness rating *t*(15) = 1.71, *p* = .109.

#### 1.1.4 Transcranial magnetic stimulation

##### Stimulation sites

The vertex was defined as the intersection between the lines connecting the nasion and the inion, and the two tragi. MNI coordinates from 10 TMS studies investigating action understanding in the lIFG were averaged, resulting in: *x* = −53, *y* = 11, *z* = 2 (Avenanti et al., 2007; Calvo‐Merino et al., 2010; Candidi et al., 2008; Catmur et al., 2011; Cattaneo, 2010; Koch et al., 2010; Pobric & Hamilton, 2006; Urgesi, Calvo‐Merino, et al., 2007; Urgesi, Candidi, et al., 2007; van Kemenade et al., 2012).

##### Stimulation parameters

rTMS pulses (two pulses per trial, frequency 10 Hz) were delivered using a Magstim Rapid2 and a 70 mm figure‐of‐eight coil (Magstim Company Ltd., UK) at 110% resting motor threshold, defined as the minimum intensity of stimulation needed to elicit motor‐evoked potentials >50 μV in the right first dorsal interosseous muscle on 5 out of 10 consecutive trials (Ellaway et al., 1998). Stimulation intensities ranged between 54% and 80% of stimulator output. rTMS pulses were delivered early (100–200 ms) or later (300–400 ms) after image stimulus onset and pulse timing was randomly distributed throughout. These pulse timings were chosen based on piloting to ensure that all stimulation pulses could be delivered before the earliest likely response time (note that any responses before the rTMS pulses were excluded from analyses; see Data Processing section).

#### 1.1.5 Procedure

Firstly, the participant's motor threshold was determined. Participants then completed 20 practice trials to familiarize them with each task, which were not repeated in the main experiment. Neuronavigation (Brainsight®, Rogue Research Inc., Montreal, QC, Canada) was then used to mark the TMS sites from a standardized structural scan onto the participant's head. This enabled the localization and monitoring of the coil position relative to these brain regions throughout the TMS sessions.

Four blocks of trials were administered, comprising 64 trials for each task at each site (action understanding task and control task at lIFG and vertex). Before each block began, participants were reminded of the task instructions and four practice trials with TMS pulses were completed in order to familiarise participants with the auditory and tactile sensations produced by the TMS pulses. Task and site order were counterbalanced across participants. For each participant, both tasks were completed at the first site of stimulation, before they were completed at the second site in the same order.

Each trial (Figure 1) commenced with a fixation cross (duration 500 ms) followed by a word phrase (action or intention) for 1000 ms. After a blank screen for 1000 ms, an image (hand or appliance) was presented until the participant responded, or for a maximum of 3000 ms. The rTMS pulses were administered at the early or late timepoint following the onset of the image. Participants indicated with a yes/no response whether the word phrase matched the image by pressing the x and m keys on the keyboard with their left and right index fingers respectively. The mapping of response key (x or m) to response (yes or no) was counterbalanced across participants and was indicated to participants via green (yes) and red (no) stickers placed over the keys. Reminder stickers were also placed at the side of the screen. A 1000 ms blank screen occurred between trials to ensure that there was a sufficient length of time between the TMS pulses, according to safety guidelines (Rossi et al., 2009). Stimuli were presented and responses recorded using Psychopy 2 (Peirce, 2007) running on a Dell Optiplex 7050 (Dell Inc., Round Rock, Texas) with a 23.8″ LED monitor (resolution 1920 × 1080, refresh rate 60 Hz).

### 1.2 Results

#### 1.2.1 Data processing

Accuracy and RT data from any trials in which participants responded before the rTMS pulses (0.3% of trials) were removed. For each participant, the mean and SD correct RTs for each cell of the design were calculated and outlying responses that were more than 2.5 SD from their corresponding mean were excluded (1.7% of trials). The proportion of correct responses and the mean RT were then calculated for each cell of the design (Table 1). For all analyses we report Bayes' Factors (BF_10_) indicating the likelihood of the observed effect under the alternative, compared to the null, hypothesis. Bayes' factors were calculated using JASP (JASP Team, 2022) default priors. In terms of interpretation, Bayes' factors BF_10_ between 1 and 3 indicate anecdotal evidence; 3–10 moderate evidence; 10–30 strong evidence; 30–100 very strong evidence; and >100 extreme evidence for the alternative hypothesis; whereas BF_10_ between 1 and 0.33 indicate anecdotal evidence; 0.33–0.1 moderate evidence; 0.1–0.033 strong evidence; 0.033–0.01 very strong evidence; and <0.01 extreme evidence for the null hypothesis (Wagenmakers et al., 2018).

**TABLE 1:** Mean and standard deviation (SD) of response times and accuracy for each cell of the design in experiment 1

Stimulation site			Left inferior frontal gyrus				Vertex			
Task	Condition	Pulse time	Response times (ms)		Accuracy (% correct)		Response times (ms)		Accuracy (% correct)	
			Mean	SD	Mean	SD	Mean	SD	Mean	SD
Action understanding	Action	100–200 ms	867.0	277.2	72.9	10.9	782.2	226.5	79.2	10.6
		300–400 ms	904.6	281.7	78.9	10.0	785.5	205.1	80.9	11.1
	Intention	100–200 ms	868.4	280.7	76.2	8.7	789.9	251.9	80.2	10.3
		300–400 ms	900.5	280.7	79.0	9.3	793.0	233.2	81.0	10.2
Control appliance	Action	100–200 ms	743.4	226.7	84.2	11.9	696.7	183.9	86.1	10.3
		300–400 ms	763.7	199.3	84.0	10.7	735.5	181.0	85.8	10.5
	Intention	100–200 ms	750.7	199.3	83.2	10.4	716.9	192.3	86.6	10.3
		300–400 ms	777.2	210.3	82.1	10.3	729.0	206.2	85.5	10.0

#### 1.2.2 Accuracy

A 2 (Site: lIFG, vertex) × 2 (Task: action understanding, control) × 2 (Condition: action, intention) × 2 (Pulse Time: early, late) repeated measures ANOVA conducted on the accuracy scores revealed a significant main effect of site, *F*(1,35) = 14.46, *p* = .001, *η* ^2^_*p*_ = 0.29, BF_10_ = 73.37. Participants were significantly more accurate for the tasks when TMS was administered to the vertex (mean = 83.2%, SD = 6.03) compared to the lIFG (mean = 80.1%, SD = 6.43). There was also a significant main effect of task *F*(1,35) = 32.92, *p* < .001, *η* ^2^_*p*_ = 0.49, BF_10_ = 1.17 × 10^12^. Participants were significantly more accurate for the control task (mean = 84.7%, SD = 7.81) than the action understanding task (mean = 78.5%, SD = 5.02). There were no other significant main effects or interactions (all *p* > .05, all BF_10_ < 0.422).

#### 1.2.3 Response times

The same ANOVA conducted on the RT data revealed significant main effects of site, *F*(1,35) = 4.96, *p* = .032, *η* ^2^_*p*_ = 0.12, BF_10_ = 7.08 × 10^6^, task, *F*(1,35) = 27.68, *p* < .001, *η* ^2^_*p*_ = 0.44, BF_10_ = 6.09 × 10^13^, and pulse time, *F*(1,35) = 9.50, *p* = .004, *η* ^2^_*p*_ = 0.21, BF_10_ = 0.103. Participants responded significantly quicker: when TMS was administered to the vertex (mean = 753.6 ms, SD = 195.2) compared to the lIFG (mean = 821.9 ms, SD = 228.9); to the control task (mean = 739.1 ms, SD = 164.8) compared to the action understanding task (mean = 836.4 ms, SD = 228.2), and to trials in which early pulses were administered (mean = 776.9 ms, SD = 194.1) compared to those where late pulses were administered (mean = 798.6 ms, SD = 191.8).

**FIGURE 2:**
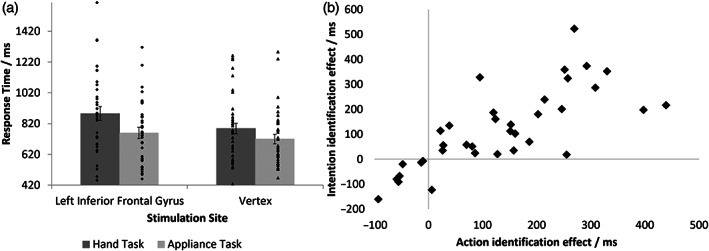
(a) Mean response times for the action understanding and control tasks when TMS pulses were administered to the lIFG and vertex, collapsed across pulse time and condition. Error bars represent standard error. Dots indicate individual participant response times. (b) Relationship between the effect of lIFG stimulation on action (*x*‐axis) and intention (*y*‐axis) identification

There was also a significant interaction between site and task, *F*(1,35) = 6.08, *p* = .019, *η* ^2^_*p*_ = 0.15, BF_10_ = 4.02 (Figure 2a). Simple effects analysis revealed that participants responded significantly slower for the action understanding task when TMS pulses were administered to the lIFG (mean = 885.1 ms, SD = 271.3) compared to when they were administered to the vertex (mean = 787.7 ms, SD = 222.8), *t*(35) = 3.06, *p* = .004, *d* = 1.04, BF_10_ = 8.96. However, there was no significant difference in RT for the control task when TMS pulses were administered to the lIFG (mean = 758.8 ms, SD = 201.6) compared to when they were administered to the vertex (mean = 719.5 ms, SD = 185.5), *t*(35) = 1.16, *p* = .255, BF_10_ = 0.332. Furthermore, this site by task interaction did not differ by condition (*F*(1,35) = 0.264, *p* = .610, *η* ^2^_*p*_ = 0.01, BF_10_ = 0.002), indicating that the effect of lIFG stimulation did not differ for action versus intention identification. That is, there was no *additional* effect of lIFG stimulation on intention identification, beyond its effect on action identification. The Bayes' factor associated with the above three‐way interaction indicates extreme evidence for the null hypothesis. This allows us to effectively rule out two possibilities. First, if the lIFG was not involved in intention understanding at all, we would have found a three‐way interaction whereby lIFG stimulation affected action identification and not intention identification. Alternatively, if the lIFG was involved *more strongly* in intention identification than in action identification we would have found a three‐way interaction in the other direction. However, the absence of a three‐way interaction indicates that the involvement of the lIFG in intention identification does not differ from its involvement in action identification.

As a further check, therefore, we tested whether any effect of lIFG stimulation on *intention* identification for hand actions remained once the effect of lIFG stimulation on *action* identification was accounted for. Specifically, we tested whether the site by task interaction found in the main ANOVA was present for the intention identification condition alone; and whether any effect remained once the effect of lIFG stimulation on action identification was included as a covariate. A two‐way ANOVA with factors of stimulation site (lIFG, vertex) and task (action understanding, control) was performed on the response times from the *intention* identification condition and revealed main effects of site, *F*(1,35) = 4.16, *p* = .049, *η* ^2^_*p*_ = 0.11, BF_10_ = 5.28, and task, *F*(1,35) = 20.28, *p* < .001, *η* ^2^_*p*_ = 0.37, BF_10_ = 118.83, and a trend toward an interaction between site and task, *F*(1,35) = 3.11, *p* = .086, *η* ^2^_*p*_ = 0.08, BF_10_ = 1.32, consistent with the main analysis. The difference in response times between the hand and appliance tasks for the *action* identification condition was then calculated for each site and a subsequent difference score between lIFG and vertex for this measure was calculated, creating a variable which represented the effect of lIFG compared to vertex stimulation on action identification for hand compared to appliance tasks. When this covariate was added into the ANOVA above, the site by task interaction was no longer significant, *F*(1,34) = 0.01, *p* = .909, *η* ^2^_*p*_ = 0.00, BF_10_ = 0.385, indicating that the effect of lIFG stimulation on response times to identify intentions from hand actions is explained by the effect of lIFG stimulation on action identification (although the strength of the evidence for the null hypothesis is anecdotal).

Finally, to test the extent to which the disruption to intention identification could be explained by the disruption to action identification, the differences in response times between the hand and appliance tasks were calculated for each condition and site, yielding action and intention identification effects for each site. These values were entered into a regression analysis to determine which of these effects predicted the intention identification effect for lIFG; that is, the extent to which lIFG stimulation disrupted response times to identify intentions. The three predictors were: the vertex intention identification effect; the vertex action identification effect; and the lIFG action identification effect. Both the vertex intention identification effect (*β* = −.073, *t* = 0.42, *p* = .680, BF_10_ = 0.508) and the vertex action identification effect (*β* = .277, *t* = 1.53, *p* = .136, BF_10_ = 1.123) were not significant predictors of the lIFG intention identification effect, whereas, as illustrated in Figure 2b, the extent to which *action* identification was disrupted by lIFG stimulation was strongly predictive of the extent to which *intention* identification was affected (*β* = .666, *t* = 5.56, *p* < .001, *R* ^*2*^ = .570, BF_10_ = 11,404.82).

### 1.3 Discussion

Experiment 1 sought to investigate the role of the lIFG in action understanding by delivering disruptive stimulation during the processing of actions and intentions performed either by human hands, or, in the control task, by appliances.

Participants responded significantly more accurately and quickly for the control task than the action understanding task. Although the control task was designed to be as difficult as the action understanding task, the nature of the stimuli is less ambiguous. Appliances are typically designed for a particular purpose and, therefore, identifying the action and intention associated with them is relatively easy. For hand actions, however, one configuration of the hand could result in the performance of several different actions and in turn relate to several different intentions (Jacob & Jeannerod, 2005). This ambiguity of hand actions is what makes “action understanding” processes so complex and is difficult to recreate with other stimuli, making direct comparisons of accuracy across tasks difficult. However it should be noted that the control task still controls for non‐specific elements of the task, such as word and image processing, decision making, and motor responses.

Participants were also more accurate and responded more quickly when TMS was applied to the vertex compared to the lIFG, providing evidence for the involvement of the lIFG in the processes measured by these tasks. The response time data also revealed a main effect of pulse time when analyzed using frequentist, although not with Bayesian, statistics. This is likely to be due to the pulses acting as an alerting trigger for participants to respond, due to the auditory and tactile sensations produced, rather than a direct effect of the stimulation.

Most importantly, the significant interaction between site and task on response times revealed that participants were significantly slower to respond to the action understanding task when TMS was applied to the lIFG compared to when it was applied to the vertex, with moderate evidence for this effect. For the control appliance task, this difference was not significant, with moderate evidence for the absence of this effect. Furthermore, the effect of lIFG stimulation did not differ across conditions: in particular there was no additional effect of lIFG stimulation on intention identification, once its effect on action identification was accounted for, with anecdotal evidence for the null hypothesis. These results, therefore, indicate a role of the lIFG in both action identification and intention identification, but that the role of the lIFG in intention identification is limited to its involvement in action identification. Finally, the regression analysis demonstrated that the more that action identification was affected by lIFG stimulation, the worse participants were at identifying the intention underlying someone else's actions, with extremely strong evidence for this effect. These results cannot be due to the fact that both the action and intention identification conditions involved processing hand actions: the regression analysis was carried out using the differences in response times for hand compared to appliance tasks. Furthermore, the vertex intention identification effect shared both word and image stimuli with the lIFG intention identification effect, but was not a significant predictor of the latter effect (anecdotal evidence for the null), in contrast to the lIFG action identification effect.

These results are consistent with previous brain stimulation studies demonstrating the role of the lIFG in processing others' actions (e.g. Avenanti et al., 2007; Candidi et al., 2008; Cattaneo, 2010; Decroix et al., 2020; Pobric & Hamilton, 2006; Reader & Holmes, 2018; Urgesi, Candidi, et al., 2007; van Kemenade et al., 2012). However, they do not fit with an account of mirror neuron function in which mirror neurons perform intention identification independently of, or in addition to, their role in action identification. Instead they suggest that, in previous studies where mirror neurons have been claimed to perform intention identification (e.g., Michael et al., 2014), they are in fact contributing to action identification, and it is this contribution to the initial action identification process which then impacts intention inference.

These results also do not provide any independent evidence for the involvement of the lIFG in intention identification, as this process was not significantly impaired by lIFG stimulation separately to its impact on action identification (and in fact there was extreme evidence for the null hypothesis for this analysis). However, Experiment 1 only stimulated one mirror neuron brain region: it could be that other mirror neuron brain regions (e.g. in parietal cortex; Fogassi et al., 2005) are contributing to intention identification. Alternatively, identification of intentions may be performed by other brain areas; a potential candidate being mentalizing areas, those involved in representing others' mental states (Saxe & Kanwisher, 2003). Therefore, Experiment 2 used functional magnetic resonance imaging (fMRI) to identify mirror neuron and mentalizing brain regions through well‐validated localizer tasks. We also measured the responses in these brain regions of interest to the identification of actions, and to the identification of intentions. If mirror neuron brain regions are involved in intention identification we should find additional responses in these regions to the identification of intentions, over and above any responses to the identification of actions. If, however, the identification of intentions is performed in mentalizing regions, we should instead find that pattern of responses in those regions of interest.

## 2 EXPERIMENT 2

### 2.1 Method

#### 2.1.1 Participants

Available funds allowed a maximum of 39 participants to be tested. This sample size was almost twice that of previous fMRI studies investigating action understanding, at the time of data collection (mean = 20.33, SD = 4.66; de Lange et al., 2008; Iacoboni et al., 2005; Libero et al., 2014; Molenberghs, Hayward, et al., 2012; Ondobaka et al., 2014; Ortigue et al., 2009; Spunt & Adolphs, 2014; Spunt et al., 2016). Thirty‐nine right‐handed native English speakers were therefore recruited via King's College London recruitment email and participated. Two participants were excluded due to excessive head movement. This resulted in 37 participants (nine male) aged 19–43 years (mean = 24.32, SD = 5.23). Although sample size was limited by funding and as such was not subject to an a priori power analysis, we note that in the region of interest analyses, this sample size provided power of .8 to detect effects of at least *d* = 0.42 at an alpha level of .05.

#### 2.1.2 Tasks

**FIGURE 3:**
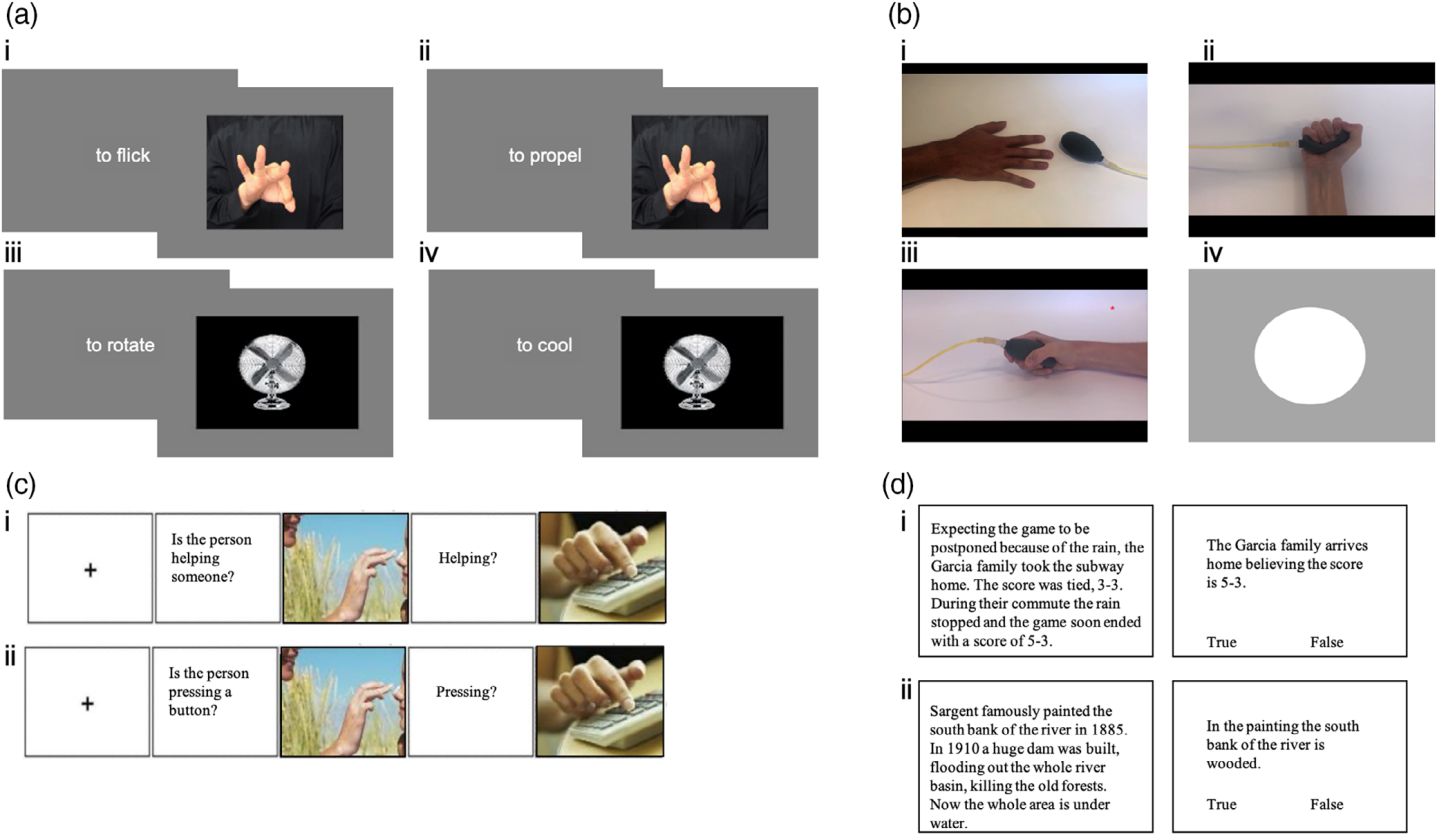
Illustrative stimuli for tasks completed in experiment 2. (a) Action understanding and control appliance tasks. (i) Hand action identification; (ii) hand intention identification; (iii) appliance action identification; (iv) appliance intention identification. (b) Mirror neuron localizer. (i) Control; (ii) observe; (iii) catch; (iv) execute. (c) Why/How localizer. (i) Why; (ii) how. (d) False belief localizer. (i) False belief; (ii) false photo

All participants completed four tasks (Figure 3) in the following order. See Supplementary Material for more details of stimuli and procedure for each task.

##### Action understanding

The action understanding and control tasks used in Experiment 1 were combined into a block design with four conditions: action and intention identification within the action understanding task (Hand Action and Hand Intention), and action and intention identification within the appliance task (Control Action and Control Intention).

##### Mirror neuron localizer

The mirror neuron localizer task was based on previous localizers (Arnstein et al., 2011; de la Rosa et al., 2016; Martineau et al., 2010; Press et al., 2012), consisting of observe, execute, control, catch, and baseline blocks.

##### Mentalizing localizer: Why/How

The hand portion of the Why/How task (Spunt & Adolphs, 2014) was used. Each trial comprised a question followed by an image of a hand action. The question varied as a function of the condition (How or Why) and referred to how the action was being performed (e.g., “is the person pressing a button?”) or to the intention underlying the action (e.g., “is the person helping someone?”).

##### Mentalizing localizer: False belief

The Why/How task contains images of actions and as such could be biased toward localizing more action‐related brain responses. Therefore, the final task was a replication of a text‐based mentalizing localizer by Dodell‐Feder et al. (2010). The stimuli comprised two sets of 10 stories requiring participants to represent false content: either about the beliefs a person holds (False Belief) or about a photograph/map/painting (False Photo).

#### 2.1.3 Procedure

All participants initially took part in a screening session, in which eligibility was assessed and the tasks were practiced within a mock scanner (see Supplementary Material). During the scanning session, task stimuli were projected onto a rear projection screen in the bore of the scanner and were viewed using a mirror attached to the head coil. See Supplementary Material for scanning parameters.

#### 2.1.4 fMRI analysis

fMRI data were analyzed using SPM12 (Wellcome Department of Imaging Neuroscience, UCL, London, UK; www.fil.ion.ucl.ac.uk/spm). See Supplementary Material for details of pre‐processing and first and second level analyses.

### 2.2 Results

See Supplementary Material for the behavioural data.

#### 2.2.1 fMRI data

##### Action understanding task

###### Action identification

**FIGURE 4:**
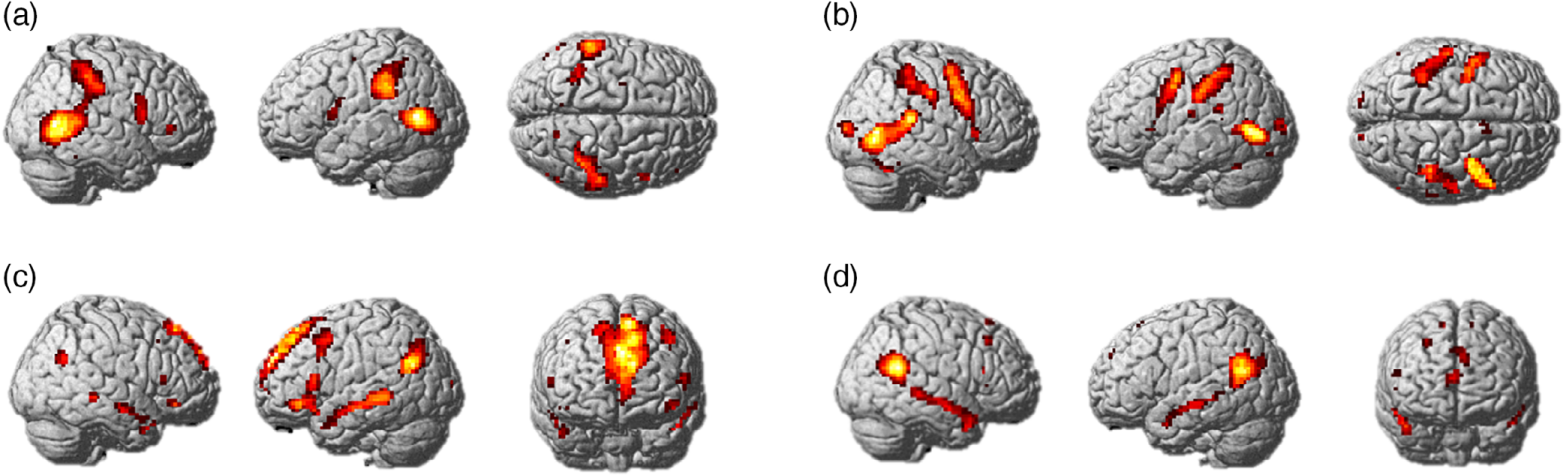
Responses to main contrasts for tasks completed in experiment 2. (a) Action identification: Hand Action > Control Action. (b) Mirror neuron localizer: Conjunction of observe > baseline and execute > baseline. (c) Why/How localizer: Why > How. (d) False belief localizer: False belief > false photo

Statistical images were created for the Hand Action > Control Action contrast in order to investigate the brain regions active during action identification, while controlling for non‐specific processes such as low‐level visual processing, decision making, and motor responses due to button presses. Activation (corrected at *p* < .05 FWE) was found bilaterally in the precuneus and IFG (pars opercularis), in the left middle occipital gyrus, inferior parietal lobule, middle frontal gyrus, superior frontal sulcus, and in the right inferior temporal gyrus, middle temporal gyrus, cuneus and IFG (pars orbitalis) (Figure 4a). The coordinates of peak activation are shown in Table S1.

###### Intention identification

In order to investigate the brain regions that were recruited during intention identification in addition to, and independent of, those recruited during action identification, statistical images were created for the contrast Hand Intention>Hand Action. Activation (corrected at *p* < .05 FWE) was found for one cluster within the left superior frontal gyrus (Table S1).

##### Mirror neuron localizer task

Mirror neuron brain regions were determined by conducting a conjunction analysis between Observe > Baseline and Execute > Baseline. As shown in Figure 4b, this revealed bilateral activation in the cerebellum, cuneus, inferior parietal lobule, supplementary motor area, precentral gyrus and laterally, in the right fusiform gyrus, posterior middle temporal gyrus, middle cingulate gyrus, and in the left middle occipital gyrus, lingual gyrus, superior temporal gyrus, supramarginal gyrus, insula, postcentral gyrus and IFG (pars opercularis).

To perform the univariate region of interest (ROI) analyses, peak coordinates from each cluster identified within the conjunction analysis were used as the centre of each ROI and spheres (radius = 10 mm) were created around these coordinates, resulting in 21 ROIs (Table S2).

##### Why/How localizer task

Statistical maps of the Why > How contrast revealed bilateral activation in the middle occipital gyrus, middle temporal gyrus, angular gyrus, posterior cingulate gyrus, middle frontal gyrus, lateral orbito‐frontal cortex and laterally in the right temporal pole, anterior superior temporal sulcus, IFG (pars triangularis) and in the left hippocampus, parahippocampal gyrus, and dorsomedial prefrontal cortex (Figure 4c).

To create the first mentalizing brain region localizer, the peak coordinates from each cluster were used as the centre of each ROI as above, resulting in 16 ROIs (Table S3).

##### False belief localizer task

The False Belief > False Photo contrast revealed bilateral activation in the TPJ, precuneus, superior frontal gyrus, medial prefrontal cortex, and laterally in the right anterior superior temporal sulcus/temporal pole, middle frontal gyrus, IFG (pars triangularis) and in the left superior temporal sulcus, caudate and dorsomedial prefrontal cortex (Figure 4d).

To create the second mentalizing brain region localizer, the peak coordinates from each cluster were used as the centre of each ROI as above, resulting in 12 ROIs (Table S4).

##### Region of interest analysis

###### Mirror neuron ROIs

To determine whether brain responses for action identification were located within mirror neuron brain regions, for each of the ROIs identified in the mirror neuron localizer task, one‐sample t‐tests were performed on the beta values for the contrast Hand Action>Control Action using the MARSBAR package in SPM12 (Brett et al., 2002). Following Bonferroni correction, nine of the ROIs showed a significant effect: left occipital middle gyrus, *t*(36) = 9.39, *p* < .001, BF_10_ = 5.984 × 10^8^, left superior temporal gyrus *t*(36) = 3.12, *p* = .023, BF_10_ = 20.47, right posterior middle temporal gyrus, *t*(36) = 12.53, *p* < .001, BF_10_ = 1.244 × 10^12^, left inferior parietal lobule, *t*(36) = 5.16, *p* < .001, BF_10_ = 4414.24, right inferior parietal lobule, *t*(36) = 6.17, *p* < .001, BF_10_ = 79,808.52, left supramarginal gyrus, *t*(36) = 3.66, *p* = .004, BF_10_ = 75.96, right precentral gyrus, *t*(36) = 3.70, *p* = .003, BF_10_ = 84.49, and two areas in left IFG (pars opercularis), *t*(36) = 3.02, *p* = .031, BF_10_ = 16.33, and *t*(36) = 3.03, *p* = .030, BF_10_ = 16.48.

To determine whether mirror neuron brain regions showed independent/additional activation for intention identification compared to action identification, one‐sample *t*‐tests were also conducted on the beta values for the Hand Intention>Hand Action contrast. No ROIs reached significance (all *t* < 1.53, all *p* > .761, all BF_10_ < 0.937, with BF_10_ < 0.227 for the two left IFG ROIs).

###### Why/How ROIs

To determine whether mentalizing brain regions showed additional activation for intention identification over and above that of action identification, one‐sample *t*‐tests were conducted on the beta values for the Hand Intention > Hand Action contrast extracted from the ROIs identified in the Why>How contrast. Following Bonferroni correction, two ROIs reached significance: the left angular gyrus, *t*(36) = 3.41, *p* = .007, BF_10_ = 40.79, and left lateral orbito‐frontal cortex, *t*(36) = 3.16, *p* = .015, BF_10_ = 22.42.

###### False belief ROIs

To determine whether brain regions associated with false belief processing showed additional activation for intention identification over and above that of action identification, one‐sample t‐tests were conducted on the beta values for the Hand Intention > Hand Action contrast extracted from the ROIs identified in the False Belief > False Photo contrast. Following Bonferroni correction, two ROIs reached significance: the precuneus, *t*(36) = 2.70, *p* = .046, BF_10_ = 7.99, and left superior frontal gyrus, *t*(36) = 3.64, *p* = .002, BF_10_ = 72.22.

### 2.3 Discussion

The aim of the present study was to investigate whether mirror neuron brain regions are involved in action and intention identification. ROI analysis of the brain regions active during the observation and execution of hand actions in the mirror neuron localizer task, revealed that action identification activated several mirror neuron brain regions, including the inferior parietal lobule, supramarginal gyrus, precentral gyrus and lIFG (pars opercularis), with evidence ranging from strong to extreme for these analyses. However, ROI analysis of the additional activation produced during intention identification revealed anecdotal to moderate evidence against the involvement of mirror neuron brain regions in this process. Instead, this activation was found in brain regions associated with two independent mentalizing brain region localizer tasks, specifically the angular gyrus, precuneus, superior frontal gyrus, and the lateral orbito‐frontal cortex, with moderate to very strong evidence for the involvement of these brain regions in intention identification.

Evidence for the role of mirror neuron brain regions in action identification supports previous findings that action identification involves mirror neuron brain regions, such as the IFG (de Lange et al., 2008; Molenberghs, Cunnington, et al., 2012; Ondobaka et al., 2014) and inferior parietal lobule (Libero et al., 2014; Ondobaka et al., 2014; Spunt & Adolphs, 2014; Spunt & Lieberman, 2012, 2013). The present experiment built on these studies by using an action understanding task in which factors such as the nature of the stimuli and abstractness of the word phrases were controlled across the action and intention identification conditions. The present study therefore supports previous findings that mirror neuron brain regions are involved in identification of the configuration of body parts involved in an observed action, and confirms that these results are not due to confounding factors such as level of abstraction (cf. Spunt et al., 2016).

The current study further demonstrates that even when these factors are controlled for, mentalizing brain regions, and not mirror neuron brain regions, respond during intention identification. This was the case not only for mentalizing regions identified using the Why/How task, which involves action stimuli; but also for regions identified using a non‐action‐based false belief task. Altogether the present study suggests that mirror neuron brain regions do not directly encode the intentions underlying observed hand actions, but instead this process relies on mentalizing brain regions.

Recent evidence suggests that mirror neuron brain regions are functionally connected to brain regions within the mentalizing network, sending configural and kinematic information to be interpreted (Cole et al., 2019; Liew et al., 2011; Mainieri et al., 2013; Van Overwalle & Baetens, 2009). In particular, when inferring others' internal states from human actions, an increase in functional connectivity between mirror neuron brain regions and the dorsomedial prefrontal cortex has been more frequently reported (Ciaramidaro et al., 2014; Cole et al., 2019; Spunt & Lieberman, 2012) than increased connectivity between mirror neuron brain regions and the TPJ (Spunt & Lieberman, 2012). In the current study, however, activation for the Hand Intention > Hand Action contrast was found within both frontal and temporoparietal ROIs, suggesting that both these brain areas process information from mirror neuron brain regions.

The limited activation produced by the intention identification condition when controlling for action identification processing could be seen as a limitation of the action understanding task. However, this contrast did reveal moderate to very strong evidence for responses in mentalizing brain regions, indicating that the failure to find such responses in mirror neuron brain regions cannot be attributed solely to the task. Instead, our results suggest that additional activation found in previous studies may be due to the influence of confounding variables, such as objects, producing greater activation that is not specific to intention identification. Due to the controlled nature of the action understanding task, the activation found within the intention identification contrast can be specifically associated with intention identification. Future studies should incorporate equally controlled measures within action understanding tasks, in order to replicate the independent activation found for intention identification within the present study.

Another future research direction would be to stimulate mentalizing brain regions during performance of the action and intention identification task, to determine the extent to which the responses in mentalizing brain regions found in Experiment 2 underlie intention identification (although the more medial areas would be difficult to target with rTMS). We chose not to stimulate an additional mentalizing brain area in Experiment 1 due to the length of the experimental session; but this would be a useful study to perform in future.

## 3 CONCLUSION

Experiment 1 illustrated that a key mirror neuron region, lIFG, contributes to action identification, but that it does not provide a separate contribution to intention identification. Furthermore, the extent to which intention identification was disrupted by lIFG stimulation was explained by the disruption to action identification, supporting the suggestion that mirror neuron contributions to intention identification are best explained via their involvement in action identification. Experiment 2 demonstrated that action identification produces responses in regions defined by an independent mirror neuron localizer, whereas intention identification produces responses in regions defined by independent mentalizing localizers.

Overall, this study indicates that while mirror neuron brain regions may contribute to action identification, there is no additional or independent contribution of these brain regions to intention identification, over and above their involvement in action identification. Instead, the findings suggest that this process requires the additional recruitment of mentalizing brain regions. Future research should investigate the functional connectivity between these two sets of brain regions in order to further understand how the configural action information encoded by mirror neuron brain regions is used to complete intention identification.

## FUNDING INFORMATION

This work was supported by the Leverhulme Trust [PLP‐2015‐019 to Caroline Catmur].

## Supporting information


**Table S1:** Peak areas of activation for the Hand Action > Control Action and Hand Intention > Hand Action contrasts in the action understanding task. All peaks survive a whole‐brain search thresholded at a voxel‐wise family‐wise error rate of *p* < .05.
**Table S2:** Peak areas of activation for the Observe>Baseline ∩ Execute>Baseline contrast in the mirror neuron localizer task. All peaks survive a whole‐brain search thresholded at a voxel‐wise family‐wise error rate of p<.05. 10mm ROIs were created around each peak coordinate. The final column indicates whether the Hand Action>Control Action contrast was significant within that ROI. Note that the Hand Intention>Hand Action contrast was not significant in any of these ROIs.
**Table S3:** Peak areas of activation for the Why > How contrast in the Why/How localizer task. All peaks survive a whole‐brain search thresholded at a voxel‐wise family‐wise error rate of *p* < .05. 10 mm ROIs were created around each peak coordinate produced by the Why>How contrast. The final column indicates whether the Hand Intention > Hand Action contrast was significant within that ROI.
**Table S4:** Peak areas of activation for the False Belief>False Photo contrast in the False Belief localizer task. All peaks survive a whole‐brain search thresholded at a voxel‐wise family‐wise error rate of *p* < .05. 10 mm ROIs were created around each peak coordinate. The final column indicates whether the Hand Intention > Hand Action contrast was significant within that ROI.

## Data Availability

Data availability statement: the data that support the findings of Experiment 1 are openly available in PsyArXiv at https://osf.io/7e5sd/?view_only=4efa8edb0b204e6e8c99885886e2c35c . The data that support the findings of Experiment 2 are available on request from the corresponding author. These data are not publicly available due to privacy or ethical restrictions.
