# Supplementary material for: Mirror neuron brain regions contribute to identifying actions, but not intentions
Source: Hum Brain Mapp. 2022 Jul 30;43(16):4901–13. doi: 10.1002/hbm.26036 (PMC9582378; doi:10.1002/hbm.26036)
Supplement: Supplementary file 1 — Table S1: Peak areas of activation for the Hand Action > Control Action and Hand Intention > Hand Action contrasts in the action understanding task. All peaks survive a whole‐brain search thresholded at a voxel‐wise family‐wise error rate of p < .05. Table S2: Peak areas of activation for the Observe>Baseline ∩ Execute>Baseline contrast in the mirror neuron localizer task. All peaks survive a whole‐brain search thresholded at a voxel‐wise family‐wise error rate of p<.05. 10mm ROIs were created around each peak coordinate. The final column indicates whether the Hand Action>Control Action contrast was significant within that ROI. Note that the Hand Intention>Hand Action contrast was not significant in any of these ROIs. Table S3: Peak areas of activation for the Why > How contrast in the Why/How localizer task. All peaks survive a whole‐brain search thresholded at a voxel‐wise family‐wise error rate of p < .05. 10 mm ROIs were created around each peak coordinate produced by the Why>How contrast. The final column indicates whether the Hand Intention > Hand Action contrast was significant within that ROI. Table S4: Peak areas of activation for the False Belief>False Photo contrast in the False Belief localizer task. All peaks survive a whole‐brain search thresholded at a voxel‐wise family‐wise error rate of p < .05. 10 mm ROIs were created around each peak coordinate. The final column indicates whether the Hand Intention > Hand Action contrast was significant within that ROI. [file HBM-43-4901-s001.docx]

Mirror neuron brain regions contribute to identifying actions, but not intentions: Supplementary Material

# Experiment 2

## Supplementary Methods

Action understanding tasks: block construction and procedure. Within each condition, eight blocks of four trials were generated with the following constraints: within each block, there was no repetition of either the word phrase or the image; and across blocks, the number of matching trials was varied such that participants could not predict the response based on the preceding trials (one block contained all matching trials; one all mismatching; two with one matching; two with two matching; two with three matching). A single block from each condition was then combined into a round of four blocks (16 trials) with the following constraints: within each round, there was no repetition of either the word phrase or the image; and across the trials within a round, half of the word phrase-image pairs were matching. The order of blocks was pseudorandomised to ensure that each block occurred within each position in a round twice, preventing participants from detecting a difference between the action and intention blocks. After each round of trials, a fixation block occurred, in which a fixation cross was presented on screen. Two pseudo-randomised orders of trials were counterbalanced across participants and response-key mappings.

An initial instruction screen was presented for 5000ms reminding participants of the response mapping, followed by a blank screen for 500ms. At the beginning of each block, an instruction screen consisting of “Hand” or “Object”, along with the reminder “Remember: Index Finger=Yes/No, Middle Finger=No/Yes”, was shown for 2000ms, followed by a blank screen for 500ms. This signalled to participants the stimuli in the upcoming block to avoid any confusion in the first trial. Each trial then commenced with a fixation cross, presented for 500ms followed by the word phrase presented for 1000ms. After a blank screen for 500ms, the image was presented for 1500ms, in which time participants were required to indicate whether the word phrase described the image, pressing the corresponding response button on a button box placed in their right hand.

For baseline blocks, participants were presented with the instructions “Rest” for 2000ms followed by a blank screen for 500ms. A fixation cross then appeared on screen for 14500ms. Participants completed eight rounds, each followed by a baseline block, in one run. Each trial lasted a total of 3550ms and therefore each block including instructions lasted 16750ms. The total time to complete this task was 11 minutes 24 seconds.

Mirror neuron localizer: stimuli and procedure. In the observe condition, participants were presented with four blocks of six videos in which a male or female hand squeezed a ball. Each video lasted 2500ms of which 250ms consisted of transitions between videos. There were six female and six male videos, in which the hand was shown from three different angles: one in which the arm entered from the bottom of the screen, three in which the arm entered from the right hand side of the screen, and two in which it entered from the left hand side of the screen. The combination and order of the stimuli within blocks was pre-determined to ensure an equal number of male and female stimuli within a block and a proportional ratio of stimuli from the three different angles across blocks.

In the execute condition, a video of a solid white circle contracting and expanding six times, once every 2500ms, was presented. Participants were equipped with the same ball as in the observe and control videos in their right hand and were required to squeeze it in time with the circle on the screen, ensuring consistency of movement across trials and participants.

In the control condition, images of the same male and female hands positioned flat next to the ball were presented while the image itself moved up and down or left and right. This controls for various stimulus aspects that are present in the observe condition: the object itself; observation of a body part; and low-level visual motion. As in the observe condition, six 2500ms videos were presented in each block, with 250ms transition time within each, and hand gender controlled for within blocks. Additionally, the position of the hand in relation to the ball and the direction of movement was controlled for across blocks.

An additional two catch blocks, one including the observe stimuli and the other the control stimuli, were included to ensure participants paid attention to the stimuli during these conditions. During these blocks, a red star appeared during one stimulus (the fourth stimulus in the observe condition and the third stimulus in the control condition) and participants were required to indicate that they saw the star by pressing either button on the button box. Finally, baseline blocks consisted of a fixation cross presented on screen for 15000ms.

Altogether the task consisted of four observe, four execute, four control, four baseline and two catch blocks. Two pre-specified orders of blocks were counterbalanced across participants. There were four rounds, each consisting of one of each block type (observe, control, execute and baseline), with the catch blocks occurring during the first and last round. Before the task began, participants were verbally instructed: when they see “observe” on the screen to have the button box in their hand, and to press either button if they observe a red star; when they see “execute” on the screen to have the ball in their hand, and that when the circle on screen shrinks they should squeeze the ball and when the circle is expanding they should release the ball. No distinction was made between the observe and control conditions, and participants were unaware of the number of catch trials. Before each block began, an instruction screen was presented for 5000ms followed by a blank screen for 500ms. For the observe, control and catch blocks these were: “Observe Hold Button Box”, for the execute block: “Execute Hold Ball”, and for the baseline condition: “Rest”. This ensured that participants had sufficient time to switch between the button box and ball if needed. The stimuli for the corresponding condition were then presented on screen, lasting 15000ms followed by another blank screen for 500ms. Each block including instructions lasted 21000ms and the total time to complete the task was 6 minutes 32 seconds.

Mentalizing localizer: Why/How. The original Why/How task used both hand and emotional stimuli, whereas in the present study, to allow a direct comparison with the action understanding task, only the hand stimuli were used. Each block comprised eight trials and started with a fixation cross (used as a baseline measure) followed by the initial question cue and the first image. Each subsequent trial in the block comprised a reminder question cue and image.

This version of the task differed from the original task in two ways: the presentation of the image stimuli was fixed at 1500ms, rather than up to 1750ms depending on participants’ responses, and the fixation cross presented at the start of each block was fixed at 6000ms rather than incorporating the differences in response time of trials within the previous block. Since Spunt and Adolphs (2014, Study 1) found a mean response time ranging from 574 to 1141ms, this timing allowed adequate time for participants to respond, while being directly comparable to the response time within the action understanding task.

The order of stimuli within blocks as well as the order of blocks were taken from Spunt and Adolphs (2014). Two eight-block rounds of the stimuli were presented, resulting in a total of eight blocks per condition (Why and How) and ensuring a comparable level of power to the original task. Two block orders were created, combining the order of the hand action blocks across the four orders in the original paper. The orders consisted of one round of stimuli presented in a repeated How-Why or Why-How block pattern, followed by a second round presented in the alternative pattern.

Before the task began, participants were verbally instructed: that they were going to see a question on screen followed by an image; that they had to indicate whether the question describes each image by pressing the corresponding buttons on the button box; and that the same question would occur for each trial within a block.

At the start of the task, participants were presented with a modified version of the instruction screen used in Spunt and Adolphs (2014), reminding participants of the trial structure, for 5000ms followed by a blank screen for 500ms. At the beginning of a block, participants were presented with a fixation cross for 6000ms followed by the question for 2100ms. After a 150ms blank screen, the image was presented for a fixed time of 1500ms, in which participants were required to respond. For the remaining seven trials within the block, a reminder cue was presented for 350ms, with a 300ms blank screen between the cue and image. The mapping of button press to response was presented below each image, as in the original task. This was counterbalanced across participants but was the same as that used for the action understanding task. The total length of a block including instructions was 19000ms, and in total the task took 6 minutes 58 seconds to complete.

Mentalizing localizer: False belief. Each story was followed by a true/false question that referred either to the situation in reality or to a false representation and participants were required to respond via a button box. There were an equal number of questions that referred to the reality and to the false representation in each condition.

Two trial orders were created: one following the original order, in which the order of stories was pseudo-randomised, and the second, in which the order of the first and second half of trials were switched and the order of stimuli in each condition was reversed. Participants were randomly allocated to one of these two trial orders.

Before the task began, participants were instructed verbally: that they were going to see a story which they had to read carefully; that they would then see a statement on screen and they had to indicate whether the statement was true for the story, using the previous response mapping.

At the start of the task, participants were presented with an instruction screen for 10000ms reminding participants of the task instructions and were then presented with an answer screen for 4000ms, in which participants were asked to indicate the correct response mapping. For each block, 1000ms of fixation was presented, followed by the story for 10000ms and then the question for 4000ms. The total task duration was 5 minutes 28 seconds.

Screening session. Participants were familiarised with the scanning environment, informed of the procedure, and practiced short versions of each task. Two rounds of the action understanding and mirror neuron localizer tasks were completed while two blocks of the Why/How and False Belief localizer tasks were completed, ensuring participants were presented with all trial types for each task. The stimuli in each practice task were not used in the MRI version of the tasks but followed the same structure and constraints. The screening session took 30 minutes to complete and on average took place 11 (± SD 9.41) days before the scanning session.

Scanning session. During the scanning session, participants were verbally reminded of the task instructions before each task began. For all tasks, participants were reminded: of the mapping of response button (index finger or middle finger) to response (yes or no), which was counterbalanced across participants, but consistent across tasks; to respond as quickly as possible while maintaining accuracy; to keep as still as possible throughout the duration of the task; and the length of the task.

fMRI parameters. MRI images were acquired with a General Electric MR750 3T scanner, using a Nova Medical 32 channel head coil and a gradient-echo echo-planar imaging (EPI) sequence with the following parameters: 41 descending horizontal slices, repetition time (TR) 2s; echo time (TE), 30ms; voxel size=3.75mm x 3.75mm, slice thickness=3.3mm; flip angle=75$^{\circ}$; Field of View (FoV)=240mm; matrix size=64×64. A three dimensional, high-resolution T1-weighted image covering the entire brain was also acquired for anatomical reference (TR=7.31ms, TE=3.02ms, voxel size=1.055 x 1.055; slice thickness=1.2mm; flip angle=11$^{\circ}$, FoV=270; matrix size=256 x 256, inversion time=400). A new run was acquired for each task and the first eight seconds were reserved for T1 equilibration. Within each run time, an additional six seconds were acquired after stimulus offset to allow for recording of the haemodynamic response.

fMRI analysis. To account for T1 equilibration, the first four scans of all runs were automatically discarded. All tasks were preprocessed according to the following steps. First, the anatomical images were segmented according to the SPM12 tissue probability maps. The functional images were then spatially realigned using a rigid body transformation and were slice-time corrected with respect to the middle slice. The structural image of each participant was then coregistered with their mean functional image. Both structural and functional images were then normalized to MNI space and resampled into 1mm^3^ and 3mm^3^ voxels respectively. Finally, the images were spatially smoothed with a 6mm Gaussian kernel (full-width at half maximum).

The data were filtered using a 128Hz high-pass filter. First level analyses were conducted by fitting a general linear model in SPM12. Alongside the six regressors representing the realignment parameters, additional regressors corresponding to individual spike movements in the data were included. For the action understanding task, one regressor was created for each experimental condition (Hand Action, Hand Intention, Control Action, Control Intention). The entire duration of the block, from when the fixation appeared until the last image disappeared, was modelled and convolved with the canonical haemodynamic response function (HRF). For the mirror neuron localizer task, one regressor was created per experimental condition (observe, execute, control, catch) as well as an additional regressor for the instruction condition. This ensured that any hand movement due to alternating between the button box and ball was not included in the baseline condition. The entire duration of the block, from the onset of the first stimulus until the final stimulus disappeared, was modelled and convolved with the canonical HRF. For the Why/How localizer task, two regressors for the How and Why conditions were created. Again, the entire duration of the block, from the onset of the first question to the offset of the final image, was modelled and convolved with the canonical HRF. Finally, for the False Belief localizer task, two regressors for the False Belief and False Photo conditions were created. The entire duration of the trial, from the onset of the story to the offset of the question, was modelled and convolved with the canonical HRF.

The second level analysis for the action understanding task was performed using a full factorial ANOVA with no independence and equal variance. Two factors (Task: hand, control; Condition: action, intention) were included, each with two levels. For the mirror neuron localizer task, a within-subject one-way ANOVA with equal variances was performed including the three conditions: observe, execute and control. For the Why/How task, a single image for each participant representing the contrast of the Why and How conditions at the first level was entered into a one-sample t-test at the second level, as in Spunt and Adolphs (2014). For the False Belief localizer task, a single image for each participant representing the contrast of the False Belief and False Photograph conditions at the first level was entered into a one-sample t-test at the second level, as in Dodell-Feder et al. (2010). All results were thresholded using a *p*<.05 family-wise error (FWE) corrected threshold at the peak level.

## Supplementary Results

### Behavioural data.

Data processing. For all four tasks, the mean and SD correct RTs for each condition were calculated for each participant, and outlying responses that were more than 2.5 SD from their corresponding mean were excluded. The proportion of correct responses and the mean RT were then calculated for each condition for each participant.

Action understanding task. A repeated measures ANOVA with factors of task (action understanding, control) and condition (action, intention) on accuracy performance revealed that participants were significantly more accurate for the control task (Mean=82.2%, SD=8.27) than the action understanding task (Mean=73.3%, SD=8.44); F(1,36)=59.36, p<.001, *η^2^_p_*=0.62. There was also a significant interaction between task and condition, F(1,36)=6.26, p=.017, *η^2^_p_*=0.15. However, simple effects analysis revealed that the simple effect of condition did not reach significance for either task.

A repeated measures ANOVA on RTs revealed participants responded significantly slower to the action understanding task (Mean=975.4ms, SD=94.5) than the control task (Mean=953.3ms, SD=90.4), *F*(1,36)=6.69, *p*=.014, *η^2^_p_*=0.16. Across tasks, participants also responded significantly faster in the action condition (Mean=957.4ms, SD=90.0) than the intention condition (Mean=971.4ms, SD=95.9), *F*(1,36)=4.59, *p*=.039, *η^2^_p_*=0.11. There was no significant interaction between task and condition.

Mirror neuron localizer task. Across participants, 98.6% (CI: 95.9 – 101.4) of catch trials were correctly identified. The execute condition was measured by applied pressure to the ball. Participants correctly executed the hand movement in 100% of blocks.

Why/How localizer task. Paired-sample t-tests indicated that there was no significant difference in accuracy performance between the How (Mean=91.1%, SD=3.88) and Why conditions (Mean=90.6%, SD=5.40), *t*(36)=0.43, *p*=.676, or in RTs between the How (Mean=881.6ms, SD=80.1) and Why conditions (Mean=892.9ms, SD=92.1), *t*(36)=1.47, *p*=.151.

False belief localizer task. Paired sample t-tests revealed that there was no significant difference in accuracy scores between the False Belief (Mean=80.0% SD=13.3) and False Photo conditions (Mean=77.6%, SD=13.8), *t*(36)=0.99, *p*=.330, however participants responded significantly slower for the False Belief condition (Mean=2416.9ms, SD=318.0) than the False Photo condition (Mean=2329.7ms, SD=304.8), *t*(36)= 2.37, *p*=.024, *d*=0.39.

### fMRI data.

Table S1 displays peak activation for the action understanding task.

Table S1

Peak areas of activation for the Hand Action>Control Action and Hand Intention>Hand Action contrasts in the action understanding task. All peaks survive a whole-brain search thresholded at a voxel-wise family-wise error rate of *p*<.05.

| **Contrast Name** | | | peak MNI coordinates | | |  |  |
| --- | --- | --- | --- | --- | --- | --- | --- |
|  | Region Name | | x | y | z | t-value | cluster size |
| **Hand Action > Control Action** | | |  |  |  |  |  |
|  | Right middle temporal gyrus |  | 51 | -64 | -1 | 14.14 | 1117 |
|  |  |  | 48 | -61 | 8 |  |  |
|  |  |  | 39 | -37 | 32 |  |  |
|  | Left middle occipital gyrus | | -51 | -70 | 5 | 13.85 | 405 |
|  | Left inferior parietal lobule | | -54 | -37 | 32 | 9.52 | 521 |
|  |  | | -36 | -43 | 41 |  |  |
|  |  | | -24 | -46 | 38 |  |  |
|  | Right IFG (pars opercularis) | | 48 | 8 | 11 | 7.53 | 83 |
|  | Right inferior temporal gyrus | | 45 | -43 | -19 | 6.36 | 8 |
|  | Right cuneus | | 15 | -91 | 2 | 6.03 | 10 |
|  | Left IFG (pars opercularis) | | -48 | 8 | 11 | 5.87 | 31 |
|  |  | | -48 | 2 | 20 |  |  |
|  | Right IFG (pars orbitalis) | | 45 | 35 | 2 | 5.74 | 20 |
|  | Precuneus | | -9 | -58 | 50 | 5.51 | 7 |
|  | Right precuneus | | 15 | -64 | 50 | 5.46 | 18 |
|  | Left middle frontal gyrus | | -24 | -10 | 50 | 5.08 | 7 |
|  | Left superior frontal sulcus | | -27 | -7 | 56 | 4.85 | 2 |
| **Hand Intention > Hand Action** | | |  |  |  |  |  |
|  | Left superior frontal gyrus | | -21 | 26 | 53 | 5.58 | 7 |

Table S2 displays the regions of interest for the mirror neuron localizer task along with an indication of which regions showed significant effects of action identification.

Table S2

Peak areas of activation for the Observe>Baseline $\cap$ Execute>Baseline contrast in the mirror neuron localizer task. All peaks survive a whole-brain search thresholded at a voxel-wise family-wise error rate of *p*<.05. 10mm ROIs were created around each peak coordinate. The final column indicates whether the Hand Action>Control Action contrast was significant within that ROI. Note that the Hand Intention>Hand Action contrast was not significant in any of these ROIs.

|  | Peak MNI coordinates | | |  |  | Significant HA > CA contrast in this ROI |
| --- | --- | --- | --- | --- | --- | --- |
| Region Name | x | y | z | t-value | cluster size |  |
| Right posterior middle temporal gyrus | 42 | -64 | 2 | 15.86 | 583 | ✓ |
|  | 60 | -34 | 17 |  |  |  |
|  | 51 | -40 | 11 |  |  |  |
| Left middle occipital gyrus | -42 | -73 | 2 | 13.73 | 261 | ✓ |
| Right cuneus | 18 | -94 | 8 | 12.26 | 98 |  |
| Right cerebellum | 30 | -52 | -22 | 11.89 | 95 |  |
|  | 30 | -61 | -22 |  |  |  |
|  | 39 | -49 | -28 |  |  |  |
| Left cerebellum | -33 | -58 | -25 | 11.45 | 34 |  |
| Right precentral gyrus | 45 | 2 | 47 | 10.31 | 423 | ✓ |
|  | 48 | 5 | 32 |  |  |  |
|  | 39 | -1 | 59 |  |  |  |
| Left cuneus | -18 | -91 | 5 | 9.62 | 87 |  |
| Left precentral gyrus | -39 | -7 | 47 | 9.40 | 206 |  |
|  | -48 | -1 | 38 |  |  |  |
|  | -54 | 5 | 35 |  |  |  |
| Left inferior parietal lobule | -42 | -31 | 41 | 8.58 | 403 | ✓ |
|  | -57 | -22 | 35 |  |  |  |
|  | -39 | -43 | 53 |  |  |  |
| Right inferior parietal lobule | 42 | -37 | 56 | 8.49 | 270 | ✓ |
|  | 51 | -22 | 38 |  |  |  |
|  | 42 | -34 | 44 |  |  |  |
| Left lingual gyrus | -15 | -85 | -16 | 7.56 | 24 |  |
| Supplementary motor area | 6 | 11 | 53 | 6.16 | 16 |  |
| Left supramarginal gyrus | -45 | -40 | 23 | 5.76 | 24 | ✓ |
| Left insula | -36 | 17 | 5 | 5.66 | 7 |  |
| Left postcentral gyrus | -60 | -16 | 20 | 5.53 | 8 |  |
| Left IFG (pars opercularis) | -54 | 14 | 5 | 5.30 | 1 | ✓ |
| Middle cingulate gyrus | 9 | 20 | 38 | 5.28 | 3 |  |
| Left IFG (pars opercularis) | -57 | 14 | 11 | 5.19 | 1 | ✓ |
| Left middle occipital gyrus | -27 | -82 | -16 | 4.97 | 1 |  |
| Left superior temporal gyrus | -48 | -43 | 14 | 4.90 | 1 | ✓ |
| Right fusiform gyrus | 45 | -46 | -22 | 4.88 | 1 |  |

*Note:* IFG: inferior frontal gyrus

Table S3 displays the regions of interest for the why/how localizer task along with an indication of which regions showed significant effects of intention identification.

Table S3

Peak areas of activation for the Why>How contrast in the Why/How localizer task. All peaks survive a whole-brain search thresholded at a voxel-wise family-wise error rate of *p*<.05. 10mm ROIs were created around each peak coordinate produced by the Why>How contrast. The final column indicates whether the Hand Intention>Hand Action contrast was significant within that ROI.

|  | peak MNI coordinates | | |  |  | Significant HI > HA contrast in this ROI |
| --- | --- | --- | --- | --- | --- | --- |
| Region Name | x | y | z | t-value | cluster size |  |
| Left dorsomedial prefrontal cortex | -12 | 56 | 26 | 14.59 | 1077 |  |
|  | -3 | 50 | 35 |  |  |  |
|  | -15 | 38 | 47 |  |  |  |
| Posterior cingulate gyrus | -3 | -46 | 29 | 13.92 | 388 |  |
| Left angular gyrus | -48 | -64 | 29 | 12.99 | 214 | ✓ |
|  | -42 | -70 | 44 |  |  |  |
| Left lateral orbito-frontal cortex | -42 | 32 | -10 | 11.87 | 181 | ✓ |
|  | -39 | 17 | -16 |  |  |  |
|  | -51 | 23 | 8 |  |  |  |
| Left middle temporal gyrus | -51 | -34 | -4 | 10.82 | 275 |  |
|  | -54 | -13 | -13 |  |  |  |
|  | -51 | -4 | -16 |  |  |  |
| Right anterior superior temporal sulcus | 57 | -10 | -16 | 9.27 | 42 |  |
|  | 51 | 2 | -22 |  |  |  |
| Right angular gyrus | 51 | -64 | 29 | 8.43 | 40 |  |
| Left middle frontal gyrus | -42 | 17 | 47 | 8.37 | 102 |  |
|  | -36 | 14 | 35 |  |  |  |
| Right temporal pole | 48 | 8 | -34 | 8.09 | 14 |  |
|  | 45 | 17 | -28 |  |  |  |
| Right lateral orbito-frontal cortex | 39 | 35 | -10 | 7.72 | 17 |  |
| Left hippocampus | -24 | -10 | -13 | 6.97 | 9 |  |
| Middle occipital gyrus | -9 | -94 | 11 | 6.91 | 15 |  |
| Right IFG (pars triangularis) | 57 | 23 | 11 | 6.21 | 10 |  |
| Right middle temporal gyrus | 54 | -34 | -4 | 6.17 | 28 |  |
| Left parahippocampal gyrus | -18 | -31 | -13 | 5.84 | 4 |  |
| Right middle frontal gyrus | 42 | 20 | 44 | 5.50 | 1 |  |

*Note:* IFG: inferior frontal gyrus

Table S4 displays the regions of interest for the false belief localizer task along with an indication of which regions showed significant effects of intention identification.

Table S4

Peak areas of activation for the False Belief>False Photo contrast in the False Belief localizer task. All peaks survive a whole-brain search thresholded at a voxel-wise family-wise error rate of *p*<.05. 10mm ROIs were created around each peak coordinate. The final column indicates whether the Hand Intention>Hand Action contrast was significant within that ROI.

|  | Peak MNI coordinates | | |  |  | Significant  HI > HA contrast in this ROI |
| --- | --- | --- | --- | --- | --- | --- |
| Region Name | x | y | z | t-value | cluster size |  |
| Right TPJ | 51 | -52 | 20 | 15.62 | 368 |  |
| Left TPJ | -51 | -58 | 20 | 12.84 | 411 |  |
|  | -42 | -52 | 20 |  |  |  |
|  | -39 | -76 | 32 |  |  |  |
| Precuneus | -6 | -55 | 35 | 12.02 | 470 | ✓ |
|  | 9 | -52 | 35 |  |  |  |
| Right anterior superior temporal sulcus/temporal pole | 51 | -4 | -19 | 11.01 | 211 |  |
|  | 57 | -10 | -13 |  |  |  |
|  | 48 | 8 | -31 |  |  |  |
| Left superior temporal sulcus | -48 | 8 | -25 | 8.73 | 112 |  |
|  | -54 | -13 | -10 |  |  |  |
|  | -48 | -37 | 2 |  |  |  |
| Dorsomedial prefrontal cortex | -6 | 50 | 32 | 7.95 | 38 |  |
| Right middle frontal gyrus | 27 | 26 | 44 | 7.83 | 22 |  |
| Medial prefrontal cortex | 3 | 56 | 14 | 7.46 | 42 |  |
| Right superior frontal gyrus | 12 | 29 | 62 | 6.31 | 7 |  |
| Left superior frontal gyrus | -12 | 26 | 62 | 6.21 | 2 | ✓ |
| Right IFG (pars triangularis) | 51 | 23 | 17 | 6.16 | 6 |  |
| Right IFG (pars triangularis) | 54 | 26 | 5 | 5.36 | 1 |  |
| Left caudate | -12 | 5 | 20 | 5.36 | 1 |  |

*Note:* IFG: inferior frontal gyrus; TPJ: temporoparietal junction

**Supplementary References**

Dodell-Feder, D., Koster-hale, J., Bedny, M., & Saxe, R. (2010). fMRI item analysis in a Theory of Mind task. *NeuroImage*, *55*(2), 705–712. https://doi.org/10.1016/j.neuroimage.2010.12.040

Spunt, R. P., & Adolphs, R. (2014). Validating the why/how contrast for functional MRI studies of theory of mind. *Neuroimage.*, *99*, 301–311. <https://doi.org/10.1016/j.neuroimage.2014.05.023>.
